# The impact of admission serum lactate on children with moderate to severe traumatic brain injury

**DOI:** 10.1371/journal.pone.0222591

**Published:** 2019-09-19

**Authors:** Yue-qiang Fu, Ke Bai, Cheng-jun Liu

**Affiliations:** 1 Department of Critical Care Medicine, Children’s Hospital, Chongqing Medical University, Chongqing, China; 2 Ministry of Education Key Laboratory of Child Development and Disorders, Chongqing, China; 3 Chongqing Key Laboratory of Pediatrics, Chongqing, China; Duke University School of Medicine, UNITED STATES

## Abstract

**Background:**

Lactate is used to evaluate the prognosis of adult patients with trauma. However, the prognostic significance of admission serum lactate in the setting of pediatric traumatic brain injury (TBI) is still unclear. We aim to investigate the impact of admission lactate on the outcome in children with moderate to severe TBI.

**Methods:**

This retrospective study was conducted in a tertiary pediatric hospital between May 2012 and Jun 2018 included children with an admission Glasgow Coma Scale (GCS) of ≤13. Two hundred and thirteen patients were included in the analysis and 45 patients died in hospital.

**Results:**

Admission lactate and glucose were significantly higher in non-survivors than those in survivors (*P* < 0.05). Admission lactate was positively correlated with admission glucose and negatively correlated with GCS in all patients (n = 213), subgroup of isolated TBI (n = 112) and subgroup of GCS ≤ 8 (n = 133), respectively. AUCs of lactate could significantly predict the mortality and were higher than those of glucose in all patients, subgroup of isolated TBI and subgroup of GCS ≤ 8, respectively. Multivariate logistic regression showed that admission lactate (Adjusted OR = 1.189; 95% CI: 1.002–1.410; *P* = 0.047) was independently associated with mortality, while admission glucose (Adjusted OR = 1.077; 95% CI: 0.978–1.186; *P* = 0.133) wasn’t an independent risk factor of death. Elevated admission lactate (> 2 mmol/L) was associated with death, reduced 14-day ventilation-free days, 14-day ICU-free days and 28-day hospital-free days.

**Conclusions:**

Admission serum lactate can effectively predict the mortality of children with moderate to severe TBI. Elevated admission lactate is associated with death, reduced ventilator-free, ICU-free, and hospital-free days. Admission serum lactate could be used as a prognostic biomarker of mortality in children with moderate to severe TBI.

## Introduction

Traumatic brain injury (TBI) is an important cause of mortality and morbidity in children [1]. Children with moderate to severe TBI were in serious condition and the hospitalization rates remain relatively unchanged over the past 20 years [2]. The early identification of patients at risk may reduce medical resource and improve outcome. In recent years, the value of serum biomarkers was investigated to determine the severity of injury and predict the outcome among patients with TBI. In previous research, we validated that early hyperglycemia could predict for in-hospital mortality in children with moderate to severe TBI [3].

In clinical practice, hyperglycemia and hyperlactatemia often occurs simultaneously in critically ill patients. Serum lactate is a by-product of anaerobic metabolism, and the level of lactate in serum can reflect the degree of tissue hypoperfusion and hypoxia. Except for one study suggesting that admission serum lactate do not predict mortality of trauma patients [4], the majority of studies showed that admission or initial serum lactate could be used to evaluate the injury severity or (and) predict prognosis in adult patients with trauma [5–19]. In addition, lactate also was an effective indicator of length of stay (LOS) [20,21]. Although lactate clearance may be a better guide to the resuscitation of the trauma patient, Dekker et al. [17] argued that a single initial lactate measurement may be a more clinically useful tool to predict mortality than the calculation of lactate clearance in adult trauma patients.

Compared with the rich evidences of lactate in adult patients with trauma, there was very few studies focused on the relationship between early lactate and trauma in children [22,23]. Ramanathan et al. [22] found that elevated admission lactate level is associated with injury and outcomes in pediatric trauma patients. Shah et al. [23] argued that prehospital lactate may improve accuracy of identifying pediatric trauma patients who need critical care, including those who have normal vital signs and GCS. Both studies showed that early lactate had a good predictive value for children with trauma [22,23]. However, the prognostic significance of admission lactate in the setting of children with TBI has not been investigated. Children’s physiology and compensatory mechanisms are not the same as adults', which may mean that previous researches on adult patients with TBI may not completely be as applicable. The aim of this study was to explore relationship between the serum lactate at admission and prognosis of children with moderate to severe TBI. Moreover, we made a comparison of predictive value between admission lactate and glucose. We hypothesized that elevated admission lactate could predict the mortality in children with moderate to severe TBI and was associated with an increased risk of in-hospital mortality, prolonged mechanical ventilation, intensive care unit (ICU) and hospital stay.

## Materials and methods

### Study design

This was a retrospective cohort study examined children with moderate to severe TBI [Glasgow Coma Scale (GCS) ≤13] admitted to ICU at the Children’s Hospital of Chongqing Medical University in Chongqing, China, from May 2012 to June 2018. This study was approved by Ethics Committee of Children’s Hospital, Chongqing Medical University (Institutional Review Board of Children’s Hospital, Chongqing Medical University). The patient records were anonymized and de-identified prior to analysis. The need for informed consent was waived by our institutional review board due to the retrospective nature of this study.

The patients were included in this study based on the established criteria, including younger than 16 years, admission GCS ≤ 13 and abnormal head computer tomography scan on admission. Exclusion criteria included the following: older than 16 years, known diabetes mellitus, patients injured more than 24 hours; known hemophilia or vitamin K deficiency; without documentation of serum lactate and glucose measurement during their stay in the emergency room.

The serum lactate and glucose levels were simultaneously measured in the arterial blood gas. The patients were given routine care for head trauma as follow. Midazolam and phenobarbital sodium were used for sedation and sufentanil for analgesia. When necessary, hyperosmolar was administered to reduce intracranial pressure. Temperature was controlled to avoid hyperthermia. Neurosurgical interventions included intracranial pressure monitor, external ventricular drains, evacuation of hematoma, dissection of necrotic brain tissues, decompressive craniectomy and reduction of separation-fracture.

### Data collection and definitions

Collected information included age, gender, body weight, admission GCS score, mechanism of injury, white blood cell (WBC), hemoglobin, platelet, prothrombin time (PT), serum sodium, serum potassium and any other injuries in addition to the head injury.

According to the 2010 Pediatric Advanced Life Support guidelines, hypotension was defined as systolic blood pressure (SBP) measured in the emergency room < 70 mmHg for infants younger than 1 year, SBP < 70 +(2 × age) mmHg in years for those patients aged from 1 to 10 and SBP < 90 mmHg for children aged 11 and older.

### Outcome

The primary outcome was in-hospital mortality. Secondary outcomes were duration of mechanical ventilation, length of stay (LOS) in the ICU, and hospital. To account for death as a competing outcome, we considered the need for mechanical ventilation as ventilator-free days with a maximum of 14 days. For the LOS, we used ICU-free days and hospital-free days with a maximum of 14 and 28 days, respectively.

### Statistical analysis

Data were analyzed using the SPSS 21.0 (SPSS Inc, Chicago, IL, USA). Shapiro-Wilk analysis was used to test whether the data were normally distributed. We described normally distributed data, non-normally distributed data and categorical data by using mean (standard deviation), median (interquartile ranges or IQR) and proportion (percentage) respectively. Comparison of two medians was performed using the Mann-Whitney *U* test, and comparison of proportions was performed using Chi-square test. The Pearson correlation was used to determine the correlation between admission lactate and admission glucose, PT, white blood cell, hemoglobin, platelet, serum sodium and serum potassium. The Spearman correlation was used to determine the correlation between the admission lactate and admission GCS. Receiver Operating Characteristic (ROC) curves (Medcalc v15.11.4, Ostend, Belgium) were used to compare the areas under the curve (AUC) for admission serum lactate and glucose as predictors of mortality in all patients, subgroup of isolated TBI and subgroup of GCS ≤ 8, respectively. Univariate logistic regression was used to determine whether initial lactate and other factors were a risk factor for mortality. Multivariate analysis was performed to determine whether admission lactate was a risk factor for mortality, adjusting for admission glucose, GCS, hypotension and hemoglobin. *P* Value less than 0.05 was considered statistically significant for all analyses.

## Results

During the study period, 288 children with moderate to severe TBI were admitted. Thirty children were excluded due to receive blood transfusion or (and) surgical treatment in other hospitals after injury. Twenty-five were excluded due to more than 24 hours after injury admitted to our hospital. Eighteen were excluded due to without lactate measurement at admission. Two were excluded due to hemophilia. Two hundred and thirteen patients were included in the analysis.

The median age, body weight and GCS at admission of the patients were 52.0 months (IQR: 20.5–86.5), 17.0 kg (IQR: 12.0–25.0) and 7 (IQR: 5–10), respectively. The median admission lactate was 1.4 mmol/L (IQR: 0.8–3.8). Comparisons of demographic and clinical severity data between the survivors and non-survivors were shown in Table 1. One hundred and sixty-eight children (78.9%) survived to hospital charge, while forty-five (21.1%) died in hospital. Admission lactate, glucose, PT and proportion of hypotension were significantly higher in non-survivors than those in survivors. Non-survivors had lower GCS scores, hemoglobin, platelet and serum potassium levels than those of survivors.

**Table 1:** Clinical and biochemical characteristics of survivor and non-survivor patients.

	All patients (n = 213)	Survivors (n = 168)	Non-survivors (n = 45)	*P* value
Age, (month), IQR	52.0(20.5–86.5)	52.0(20.3–86.0)	49.0(20.0–90.5)	0.962
Weight, (kg), IQR	17.0(12.0–25.0)	18.0(11.1–25.0)	15.0(12.0–23.8)	0.679
Gender, n (%)				0.531
Male	127(59.6%)	102(60.7%)	25(55.6%)	
Female	86(40.4%)	66(39.3%)	20(44.4%)	
GSC, IQR	7(5–10)	8(6–11)	4(3–6)	<0.001
Associated injuries, n (%)	101(47.4%)	74(44.1%)	27(60.0%)	0.057
Hypotension, n (%)	45(21.1%)	18(10.7%)	27(60.0%)	<0.001
Lactate (mmol/L), IQR	1.4(0.8–3.8)	1.1(0.7–2.4)	4.7(2.95–7.7)	<0.001
Glucose(mmol/L), IQR	7.4(5.5–12.3)	6.45(5.2–9.2)	14.9(10.1–19.3)	<0.001
WBC (×10^9^/L), IQR	17.19(12.9–21.3)	17.32(13.1–21.1)	18.84(12.4–22.6)	0.886
Hemoglobin (g/L), IQR	98.0(82.5–110.5)	100.0(87.25–113.0)	85.0(68.5–102.0)	<0.001
Platelet (×10^9^/L), IQR	253.0(192.5–310.5)	260.0(206.5–321.8)	200.0(146.5–270.5)	0.001
Sodium(mmol/L), IQR	137.7(135.1–140.7)	137.9(135.2–140.7)	136.8(134.1–139.9)	0.293
Potassium (mmol/L), IQR	3.7(3.25–4.12)	3.7(3.30–4.1)	3.4(3.10–3.8)	0.010
PT (s), IQR	13.7(12.6–16.7)	13.4(12.6–14.8)	18.0(15.5–24.9)	<0.001
Mechanism of injury, n (%)				0.569
Road traffic accident	87(40.8%)	71(42.3%)	16(35.6%)	
Fall	94(44.1%)	71(42.3%)	23(51.1%)	
Other	32(15.0%)	26(15.5%)	6(13.3%)	

Data shown are number (percentage) or median (interquartile range). GCS**-** Glasgow Coma Scale; IQR- interquartile range; PT- Prothrombin time; WBC-white blood cell

Univariate and multivariate logistic regression analyses were performed to determine whether admission lactate was independently associated with mortality (Table 2). We found that admission lactate [adjusted odds ratio (OR) = 1.189; 95% confidence interval (CI): 1.002–1.410; *P* = 0.047] and GCS (adjusted OR = 0.678; 95% CI: 0.532–0.864; *P* = 0.002) were independent risk factors for the mortality when we controlled for the other variables. However, we showed admission glucose (adjusted OR = 1.077; 95% CI: 0.978–1.186; *P* = 0.133) was not an independent risk factor for mortality in this model.

**Table 2:** Univariate and multivariate logistic regression to identify risk factors at admission related to mortality.

variable	Univariate logistic regression Unjusted OR (95% CI)	*P* value	Multivariate logistic regression Adjusted OR (95% CI)	*P* value
Lactate	1.540(1.328 to 1.786)	<0.001	1.189(1.002 to 1.410)	0.047
GCS	0.509(0.406–0.638)	<0.001	0.678(0.532 to 0.864)	0.002
Glucose	1.260(1.172 to 1.355)	<0.001	1.077(0.978 to 1.186)	0.133
Hypotension	12.500(5.781 to 27.029)	<0.001	2.642(0.941 to 7.421)	0.065
Hemoglobin	0.969(0.953 to 0.984)	<0.001	0.996(0.974 to 1.019)	0.750

CI- confidence interval; GCS**-** Glasgow Coma Scale; OR**-** odds ratio

As showed in Table 3, correlation analysis revealed that admission lactate was positively correlated with admission glucose (all *P* < 0.001) and PT (all *P* < 0.001) in all patients, subgroup of isolated TBI and subgroup of GCS ≤ 8, respectively. Moreover, admission lactate was negatively correlated with admission GCS (all *P* < 0.001) and hemoglobin (*P* < 0.001, *P* = 0.002 and *P* < 0.001, respectively) in all patients, subgroup of isolated TBI and subgroup of GCS ≤ 8, respectively.

**Table 3:** Correlation between admission glucose, GCS, PT, hemoglobin and lactate in all patients, subgroup of isolated TBI and subgroup of GCS ≤ 8.

	Admission lactate		
	All patients(n = 213)	Isolated TBI (n = 112)	GCS ≤ 8 (n = 133)
Glucose	rho = 0.602; *P*<0.001	rho = 0.510; *P*<0.000	rho = 0.556; *P*<0.001
GCS	rho = -0.542; *P*<0.001	rho = -0.540; *P*<0.000	rho = -0.457; *P*<0.001
PT	rho = 0.571; *P*<0.001	rho = 0.499; *P*<0.000	rho = 0.573; *P*<0.001
Hemoglobin	rho = -0.390; *P*<0.001	rho = -0.293; *P* = 0.002	rho = -0.429; *P*<0.001

GCS**-** Glasgow Coma Scale; PT**-** prothrombin time; TBI**-** traumatic brain injury

**Fig 1:**
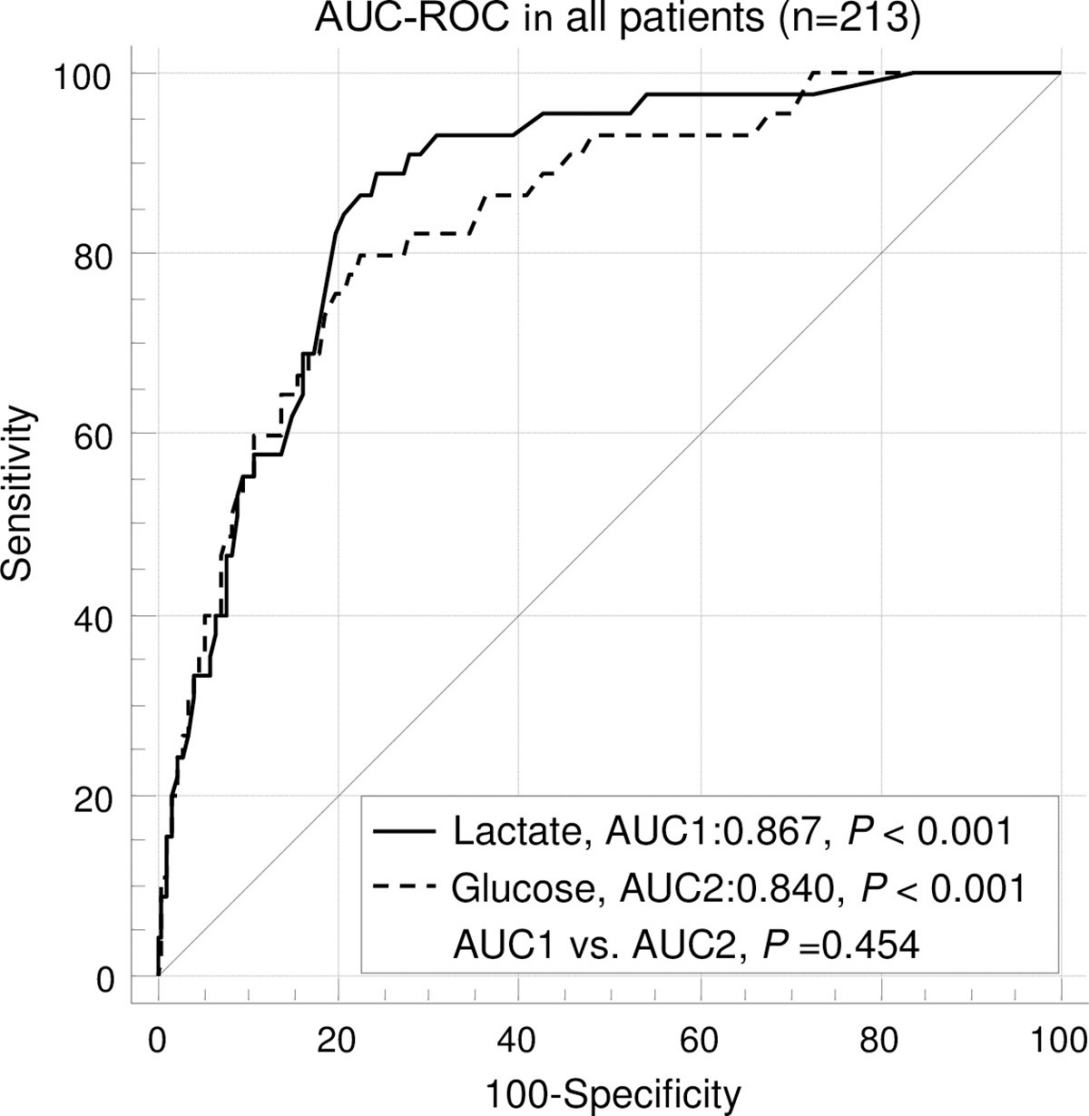
ROC curve analyses of mortality in all patients. The AUC of admission lactate was not significantly different from the AUC of admission glucose. AUC- area under the curve; GCS- glasgow coma scale; ROC- receiver operating characteristic.

**Fig 2:**
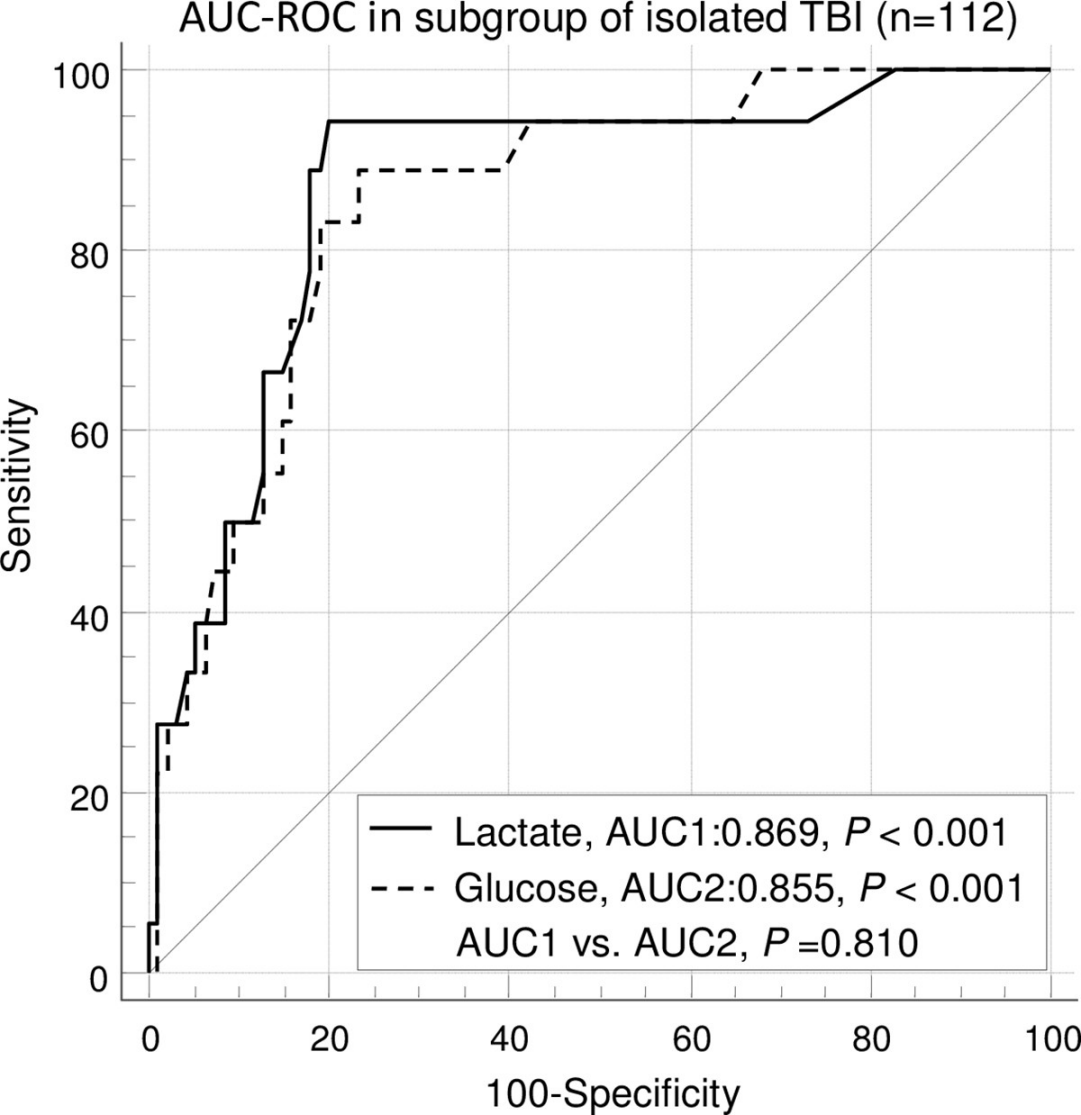
ROC curve analyses of mortality in subgroup of isolated TBI. The AUC of admission lactate was not significantly different from the AUC of admission glucose. AUC**-** area under the curve; GCS**-** glasgow coma scale; ROC**-** receiver operating characteristic.

**Fig 3:**
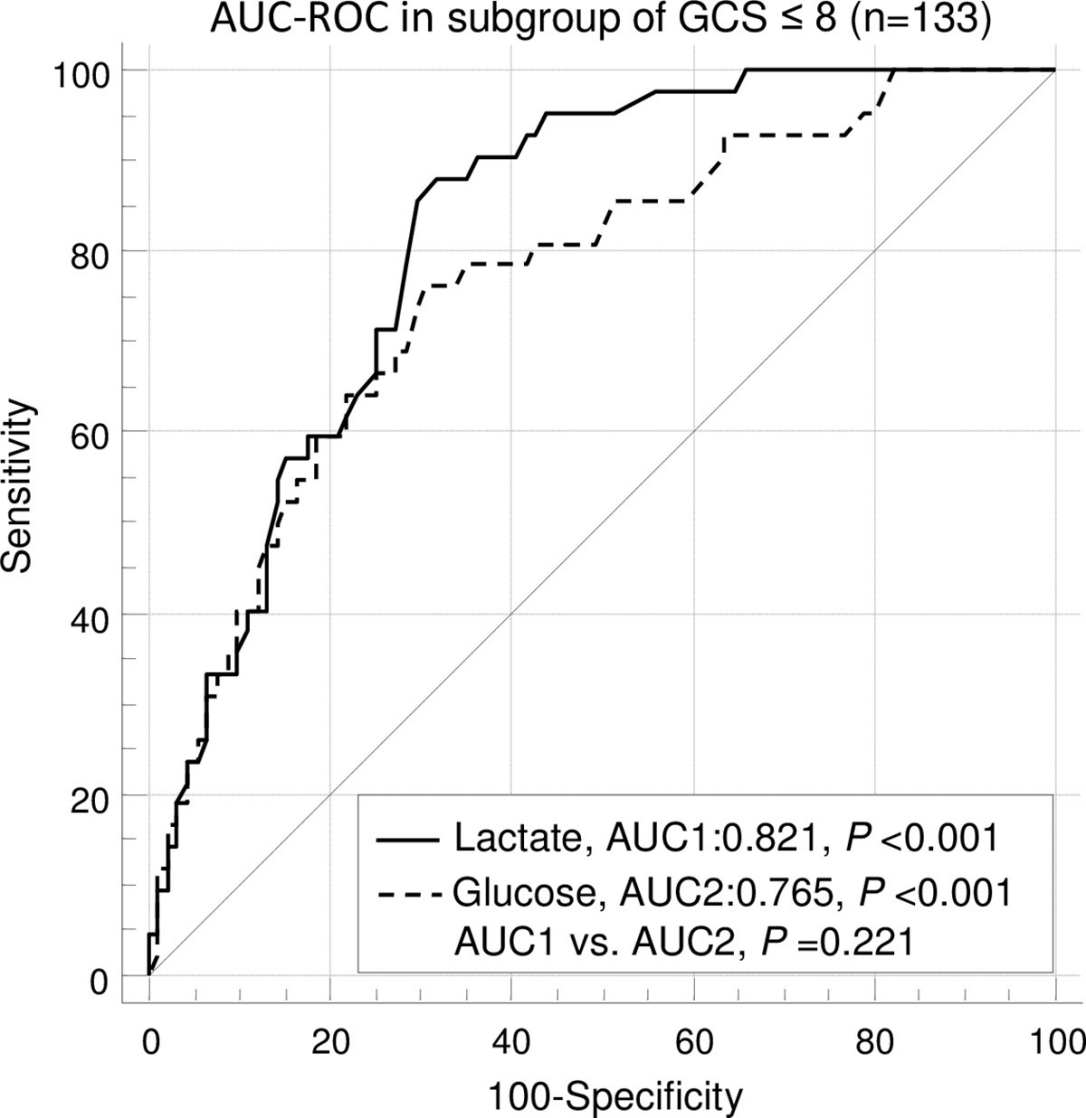
ROC curve analyses of mortality in subgroup of GCS ≤ 8. The AUC of admission lactate was not significantly different from the AUC of admission glucose. AUC**-** area under the curve; GCS**-** glasgow coma scale; ROC**-** receiver operating characteristic.

ROC curve analysis (Fig 1) showed that admission lactate (AUC 0.867, 95% CI: 0.814–0.909, *P* < 0.001) and glucose (AUC 0.840, 95% CI: 0.783 to 0.886, *P* < 0.001) were significantly effective predictors of mortality in children with moderate to severe TBI (Fig 1). In addition, AUCs of admission lactate and glucose were both statistically significant in subgroup of isolated TBI and subgroup of GCS ≤ 8 (Figs 1 and 2). The AUCs of admission lactate were larger than those of admission glucose in all patients, subgroup of isolated TBI and subgroup of GCS ≤ 8, respectively (Figs 1–3). However, the differences were not statistically significant.

The outcomes of all patients with lactate > 2.0 mmol/L and ≤ 2.0 mmol/L were presented in Table 4. Those with a level of less than or equal to 2 mmol/L had mortality of 3.2%, compared with a mortality that was more than fourteen times higher (46.6%) in those with an admission lactate of greater than 2 mmol/L. This difference was statistically significant (*P* < 0.001). Compared with patients with admission lactate ≤ 2.0 mmol/L, the duration of mechanical ventilation and ICU stay was significantly longer, while the hospital LOS was less in patients with lactate > 2.0mmol/L. To account for death as a competing outcome, we found that the 14 ventilation-free, 14 ICU-free and 28 hospital-free days were all less in patients with lactate > 2.0 mmol/L versus patients with lactate ≤ 2.0 mmol/L.

**Table 4:** Clinical outcomes based on admission lactate level for patients with GCS ≤ 13.

	Lactate ≤ 2mmol/L (n = 125)	Lactate > 2mmol/L (n = 88)	*P* values
Death, n (%)	4 (3.2%)	41 (46.6%)	<0.001
LOS of hospital (day), IQR	20.0(12.0–29.5)	12.0(1.0–35.5)	0.014
28-day hospital-free days, IQR	8.0(0–15.5)	0 (0–0)	<0.001
LOS of ICU (day), IQR	1.0(1.0–3.0)	4.0(1.0–9.0)	<0.001
14-day ICU-free days, IQR	13.0 (10.5–13.0)	0 (0–8.5)	<0.001
Ventilation days, IQR	1.0(1.0–1.0)	2.0(1.0–6.0)	<0.001
14-day ventilation-free days, IQR	13.0(12.0–13.0)	1.5(0–11.75)	<0.001

Data shown are number (percentage) or median (interquartile range). GCS**-** Glasgow Coma Scale; ICU**-** intensive care unit; IQR- interquartile range; LOS**-** length of stay;

A stratified analysis of admission lactate and outcome in patients with GCS ≤ 8 was performed (Table 5). There was a significant difference in the proportion of patients who died between the two groups (lactate ≤ 2mmol/L vs. lactate > 2.0 mmol/L: 5.4% vs. 50.6%; *P* < 0.001). There were still significant differences in 14 ventilator-free days, 14 ICU-free days and 28 hospital-free days between patients with lactate > 2.0 mmol/L versus those with lactate ≤ 2.0 mmol/L at admission.

**Table 5:** Clinical outcomes based on admission lactate level for patients with GCS ≤ 8.

	Lactate ≤ 2mmol/L (n = 56)	Lactate > 2mmol/L (n = 77)	*P* values
Death, n (%)	3(5.4%)	39(50.6%)	<0.001
LOS of hospital (day), IQR	22.0(13.0–53.75)	12.0(1.0–35.0)	<0.001
28-day hospital-free days, IQR	0.5(0–11.75)	0 (0–0)	<0.001
LOS of ICU (day), IQR	2.0(1.0–4.75)	4.0(1.0–10.5)	0.027
14-day ICU-free days, IQR	11.5 (9.0–13.0)	0 (0–6.0)	<0.001
Ventilation days, IQR	1.0(1.0–2.0)	3.0(1.0–7.0)	0.001
14-day ventilation-free days,IQR	13.0(12.0–13.0)	0(0–9.5)	<0.001

Data shown are number (percentage) or median (interquartile range). GCS**-** Glasgow Coma Scale; ICU**-** intensive care unit; IQR- interquartile range; LOS**-** length of stay;

## Discussion

Lactate is known as a marker of the metabolic stress response and elevated lactate is associated with increased mortality in critically ill patients. However, the relationship between admission lactate level and prognosis of children with TBI is still unknown. In this study, we find that admission lactate and glucose of non-survivors are significantly higher than those of survivors in children with moderate to severe TBI. Admission lactate is positively correlated with admission glucose and PT and negatively correlated with admission hemoglobin and GCS, which indicate that high levels of admission lactate is associated with high serum glucose, coagulopathy, anemia and severity of trauma. It implicates that the admission serum lactate is closely consistent with the severity of the disease in children with moderate to severe TBI.

Univariate analysis found statistically significant difference in admission lactate, glucose, GCS, hemoglobin, and proportion of hypotension between non-survivor group and survivor group. After adjusting for the other variables, multivariate logistic regression analysis showed that admission lactate was an independent risk factor for the mortality in children with moderate to severe TBI. However, admission glucose was not an independent risk factor for the mortality. The difference from our previous study [3] may be caused by different sample sizes and different parameters setting of the regression model.

For the last decade and more, some studies had evaluated the effectiveness of lactate in predicting mortality in adult trauma patients [6, 11, 17]. Parsikia et al. [6] found initial serum lactate can predict mortality of adult trauma patients whose interval between injury and arrival was within 24 h. Gale et al. [11] showed initial lactate better predicted in-hospital mortality than initial base deficit in patients with severe blunt trauma. In the field of adult brain injury, the relationship between initial lactate and TBI has been also explored. Dekker et al. [17] showed that the prognostic value of initial lactate and lactate clearance for in-hospital mortality were not found to differ between isolated TBI, polytrauma with TBI, and trauma without TBI. However, the relationship between admission serum lactate levels and mortality in children with TBI is not explored.

Using ROC analysis, our study shows that admission lactate is a reliable indicator of those who are at the risk of in-hospital death in children with moderate to severe TBI. The area under the curve is 0.867 in children with moderate to severe TBI, which indicates admission lactate is a significant predictor of mortality. Moreover, admission lactate can also predict the mortality of subgroup of isolated TBI and subgroup of severe TBI. Glucose at admission was also a very useful biomarker predictor of outcome in children with TBI [24]. Sammour et al. [25] showed that glucose at admission was the better biochemical predictor of mortality compared to lactate at admission (ROC area 0.845 and 0.716, respectively) in clinically severely injured trauma patients. Although there are no significant differences in statistics, in the present study, the AUCs of admission lactate are larger than those of admission glucose in children with moderate to severe TBI, moderate to severe isolated TBI and severe TBI, respectively. This implicates that lactate at admission may be superior to glucose in predicting mortality in children with moderate to severe TBI.

In recent years, some studies found that the brain extracellular lactate served as an alternative energy substrate to glucose for normal or injured neurons in TBI [26, 27]. However, Lama et al. [28] indicated that high brain extracellular levels of lactate might be harmful. Stefani et al. [29] showed that cerebrospinal fluid level of lactate had 100% of sensitivity and 85.71% of specificity (AUC: 0.8810, 95% CI, CL: 54.55–98.08%) with a cutoff of > 4.650 mmol/L to predict brain death in TBI patients. Meierhans et al. [30] showed arterial lactate above 2 mmol/L was associated with increased brain lactate and decreased brain glucose in patients with severe TBI. In addition to reflecting tissue hypoperfusion and hypoxia, this may be one new reason to explain the potential mechanism that elevated serum lactate is associated with poor outcome of TBI. In the present study, we find that the mortality rate of children with admission lactate > 2 mmol/L is more than 14 times higher than that of children with admission lactate ≤ 2 mmol/L. Therefore, children with moderate to severe TBI, whose admission lactate > 2 mmol/L, should be given more critical care and correctly assessed as to the severity of their injuries. Timely and effective medical treatment and/or neurosurgical intervention after admission may reduce the mortality of these patients. According to the results of adult studies [28– 30], we speculate that children with moderate to severe TBI, whose serum lactate is > 2 mmol/L, may have increased brain lactate and decreased brain glucose. This probably causes energy metabolism disorder in brain cells, which lead to secondary brain injury and contribute to the death of patients. Potential mechanism of high serum lactate affecting brain cell metabolism in children with trauma is worth exploring.

Admission lactate was associated with LOS in adult trauma patients [20,21]. In present study, we find that 14 ventilation-free, 14 ICU-free and 28 hospital-free days of the patients with initial lactate > 2.0 mmol/L are less than those of the patients with initial lactate ≤ 2.0 mmol/L. Therefore, we consider that elevated admission lactate is associated with death, reduced ventilator-free, ICU-free, and hospital-free days in children with moderate to severe TBI.

The close relationship between initial lactate elevation and the outcome in children with trauma was indicated in two studies [22,23]. Rajesh et al. [22] showed admission lactate over 4.7 mmol/L was strongly suggestive of severe injury in pediatric trauma patients. Shah et al. [23] reported that prehospital lactate level was higher in pediatric trauma patients who required critical care, including those who had normal prehospital vital signs and GCS. In the present study, we demonstrate that elevated serum lactate at admission is predictive of increased in-hospital mortality in children with moderate to severe TBI. We also show that the predictive power of lactate may be better than that of glucose, although it has no statistical significance. In addition, admission lactate is independently associated with mortality. We consider that measurement of serum lactate levels at admission in children with moderate to severe TBI may potentially be useful as a predictor of mortality, which may help clinician to better assess the severity and optimize decision-making process in children with moderate to severe TBI.

There are several limitations to this study. Firstly, this retrospective study was limited by factors that are inherent to the retrospective analysis and interpretation of data. Secondly, it is a single-center retrospective study and the sample size of this study is relatively small. However, it may be the first clinical study concerning the predictive value of serum lactate at admission in children with TBI. Thirdly, we only collected lactate at admission in this study. But we did not include lactate clearance and couldn’t assess the impact of lactate changes on the prognosis of children with TBI. Odom et al. [31] indicated that both initial lactate and lactate clearance at 6 hours independently predict death in adult trauma patients. However, the predictive value of lactate clearance in children with TBI is still unknow and further research is need.

## Conclusions

Our study suggests that serum lactate at admission is a useful biomarker for children with moderate to severe TBI. Serum lactate at admission is an independent risk factor for mortality and associated with decreased ventilation-free, ICU-free and hospital-free days in children with moderate to severe TBI.

## Supporting information

S1 Checklist STROBE statement—Checklist of items that should be included in reports of observational studies. (DOCX)
